# Age- and sex-specific impact on the progression of low-risk papillary thyroid carcinoma under active surveillance: a meta-analysis

**DOI:** 10.3389/fonc.2025.1547345

**Published:** 2025-06-18

**Authors:** Yang Wang, Li Wang

**Affiliations:** ^1^ Center for Endocrinology and Metabolic Diseases, The People’s Hospital of Liaoning Province, Shenyang, China; ^2^ Department of Endocrine and Metabolism, Zhuhai People’s Hospital (Zhuhai Hospital Affiliated with Jinan University), Zhuhai, China

**Keywords:** active surveillance, papillary thyroid cancer, oncologic outcomes, aging, meta-analysis

## Abstract

**Background:**

The global incidence of papillary thyroid carcinoma (PTC) is increasing significantly. In response, active surveillance (AS) has been promoted for Low-risk PTC owing to the absence of associated mortality. However, the association between age, sex, and risk of tumor progression remains unclear. This study aimed to assess the age- and sex-specific Impact on the progression of low-risk PTC.

**Methods:**

PubMed and Web of Science Core Collection were searched for articles published up to 1 January 2024. Articles reporting patients with PTC undergoing AS were included. Studies involving patients who underwent total or partial thyroidectomy or radiofrequency ablation were excluded. Random- and fixed-effect models were applied to obtain pooled proportions and 95% CIs.

**Results:**

A total of 972 unique citations were screened and 39 full-text articles were reviewed, including eight cohorts. The mean or median age ranged from 41.5 to 53.1 years, with a predominant inclusion of female patients (76.39%–87.80%). The pooled risk ratio for tumor progression (a growth of 3 mm or more in maximal diameter or lymph node metastasis) in older adults (aged over 30–50 years) compared with younger individuals was 0.58 (95% CI, 0.47–0.71; 4,725 patients, six studies). However, for male patients, the pooled risk ratio for tumor progression compared with female individuals was 1.11 (95% CI, 0.64–1.93; 4,916 patients, six studies).

**Conclusion:**

This meta-analysis suggests that advanced age may be associated with a lower risk of progression of papillary thyroid microcarcinomas during active surveillance. No significant differences were observed between sexes.

## Introduction

Over the past two decades, the global incidence of thyroid cancer has increased rapidly (1), with PTC being the most common subtype, accounting for approximately 85% of cases (2). The World Health Organization defines PTC with a maximum diameter of ≤10 mm as papillary thyroid microcarcinomas (PTMC) (3), and autopsy studies have detected PTMC in 5%–36% of the population (4). This increase is partly attributed to advancements in diagnostic techniques such as high-resolution ultrasonography (US) and fine-needle aspiration biopsy (FNAB).

PTC, particularly in its microcarcinoma form (PTMC), generally has excellent prognosis. Patients with PTC exhibit a 10-year overall survival rate exceeding 95% (5), whereas those with PTMC have a 10-year disease-specific survival rate of >99% (6). Although most guidelines recommend total or partial thyroidectomy with therapeutic central and lateral lymph node dissection as the first-line treatment for PTMC (7, 8), these surgical interventions are associated with significant costs and a reduced quality of life (9, 10).

AS and thermal ablation have been proposed as alternative management strategies to mitigate overdiagnosis and overtreatment. AS, initially developed for low-risk prostate cancer (11), was introduced for low-risk PTC by Professor Ito and his team at Kuma Hospital, Japan (12). Over time, the concept of observing very low-risk thyroid cancers under AS has gained acceptance among both patients and clinicians.

Since 2010, numerous medical institutions have established cohorts to study AS in low-risk PTC patients. By 2020, outcomes from these cohorts with follow-up periods ranging from 5 to 10 years have been progressively published (13–20). However, the influence of aging and gender on the risk of PTC progression during AS remains unclear, with conflicting findings reported in literature (21, 22).

This study aimed to evaluate the age- and sex-specific impact of AS on tumor progression in low-risk PTC through a comprehensive meta-analysis.

## Materials and methods

This systematic review and meta-analysis was performed according to an updated version of The Preferred Reporting Items for Systematic Reviews and Meta-Analyses (PRISMA) statement published in 2020 (23).

### Search strategy

A comprehensive search of the PubMed and Web of Science Core Collection (WOSCC) databases was conducted to identify original literature reporting on patients undergoing AS for PTC.

The following search terms were used: ((“active surveillance”) OR (observation)) AND ((papillary thyroid carcinoma) OR (papillary thyroid microcarcinoma) OR (papillary microcarcinoma of the thyroid) OR (thyroid microcarcinoma)). No starting date was specified, and the literature search was updated on 1 January 2024. Only English language publications were included in this study. Relevant article bibliographies were scrutinized for any additional suitable articles. The exclusion criteria included case reports, letters, conference abstracts, reviews, meta-analyses, guidelines, study protocols, and statements. Patients who underwent total or partial thyroidectomy and radiofrequency ablation were also excluded from the study. Three independent reviewers screened all the unique citations for relevance, reviewed the full-text articles, and reached a consensus.

### Data extraction

Two reviewers independently extracted data using standardized forms: (1) Characteristics of the included studies: institution, country of origin, authors, publication year, study design, follow-up duration, and funding information. (2) Characteristics of study participants: patient numbers, mean age, male-to-female ratio, largest primary tumor dimension, prevalence of thyroid hormone used during AS, inclusion of multifocal papillary thyroid cancer, and larger PTCs (>10 mm). (3) Clinical outcomes of patients undergoing AS: increase in maximal nodule diameter, development of new cervical lymph node metastasis (LNM) on ultrasound, and suspected distant metastasis. A consensus on the extracted data was achieved through discussion.

### Statistical analysis

Continuous variables were described using mean and standard deviation or median and interquartile range (IQR), whereas categorical variables were presented as frequencies with percentages. The meta-analysis results, expressed as risk ratios (RR) with 95% confidence intervals, were used. Heterogeneity between studies was assessed using I² statistics. If no statistical proof of heterogeneity (I² <50%) was found, a fixed- effects model was applied; otherwise, a random- effects model was used. A meta-analysis was conducted using Review Manager (RevMan) version 5.3.

## Results

### Systematic literature search

**Figure 1:**
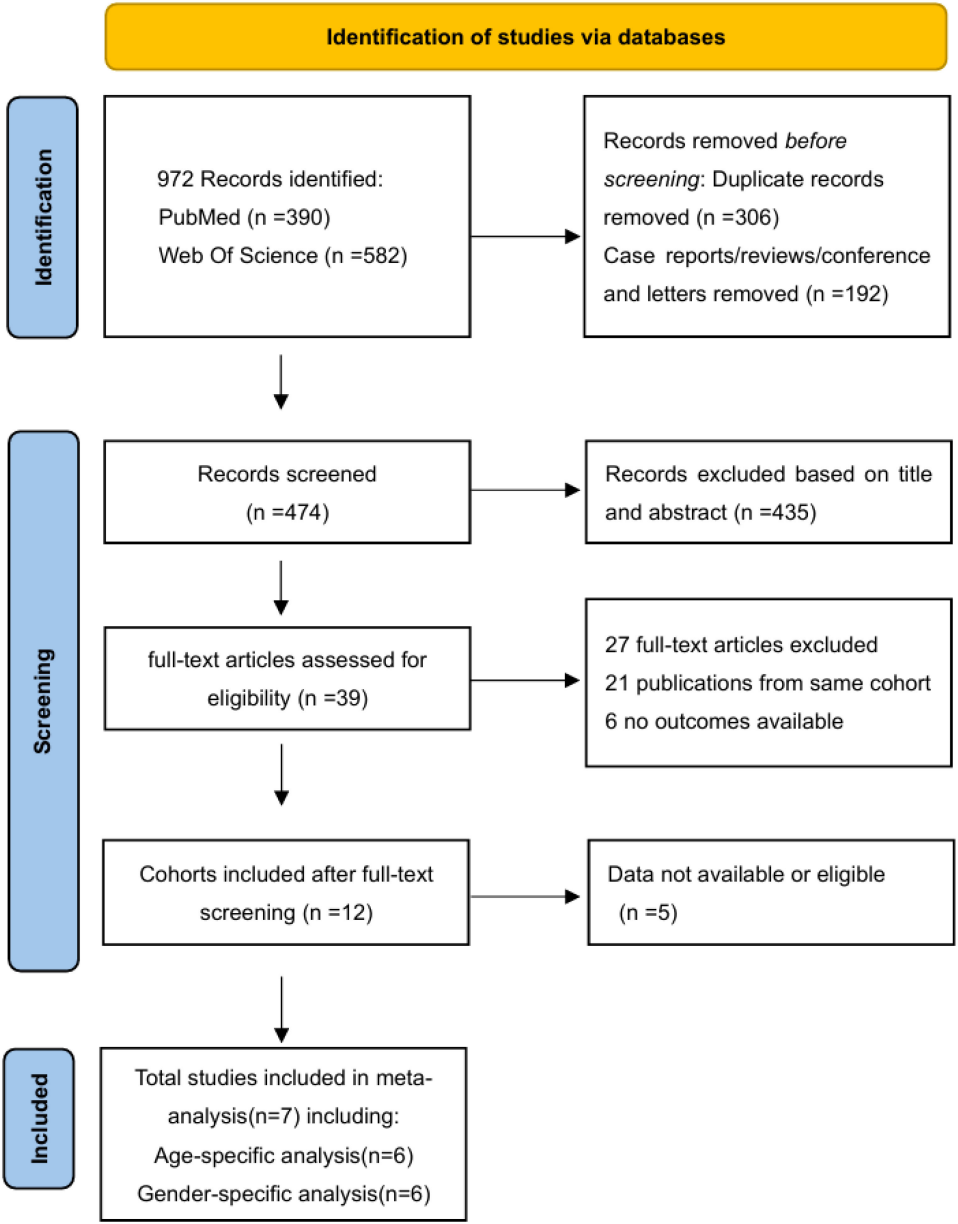
PRISMA flow diagram of literature search and study selection. A total of 972 records were identified from databases, with 306 duplicates removed. After screening titles/abstracts, 39 full-text articles were assessed for eligibility, and seven studies met inclusion criteria for quantitative synthesis.

A total of 972 publications were identified in the search updated until 1 January 2024, comprising 390 from PubMed and 582 from the Web of Science Core Collection (WOSCC). After removing 306 duplicates and excluding 192 non-original studies based on titles and abstracts, 39 full-text articles were assessed for eligibility. After careful selection, 21 publications were excluded owing to potential overlapping patient cohorts, considering author names, time periods of patient inclusion, and affiliations. In this process, priority is given to the most recent and abundant studies. Additionally, six studies were excluded due to unavailable outcomes, and four were excluded due to the absence of age or sex subtypes. Finally, seven studies were deemed eligible for the meta-analysis (13–20). Among these, six studies were included in the age-specific analysis (14–19) and six were included in the sex-specific analysis (13–15, 18–20) (**Figure 1**).

### Description of the included original studies

**Table 1** presents the characteristics of the studies included. All studies originating in Japan, Korea, China, and Argentina were published after 2020. Two studies were conducted in systematically well-managed cohorts, including the earliest and longest (1993–2019) cohort initiated by Ito et al. at Kuma Hospital (18) (Japan) and the Multicenter Prospective Cohort Study of Active Surveillance on Papillary Thyroid Microcarcinoma (MAeSTro) (NCT02938702) from Korea (17, 20). Notably, these two studies included cohorts with more than 1,000 participants. Studies in China had shorter median follow-up periods (12 and 28.5 months) (13, 19), while others had mean or median follow-up periods ranging from 41.4 months to 7.6 years (14–18, 20).

**Table 1:** Characteristics of the included studies.

Source	Country (institution)	No. of participants	Study design	Follow-up duration^a^	Funding
Hu et al. (13)	China (the First Affiliated Hospital with Nanjing Medical University)	386 patients older than 18 years with thyroid nodules <1.0 cm for the largest diameter and without clinical symptoms or known metastases were evaluated from January 2015 to December 2019, 212 patients were enrolled for AS;	Retrospective study	The median follow-up duration was 12.0 (6.0–60.0) months	None reported
Jin et al. (14)	Korea (Asan Medical Center, Samsung Medical Center, and Seoul St. Mary’s Hospital)	383 PTMC patients is screened after cytopathologically being diagnosed between 2002 and 2017, 326 PTMC patients undergoing long-term AS, followed up ≥1 y;	Cohort study	The median follow-up duration was 4.9 (3.4–6.3) years	Korea Health Technology R&D Project through the Korea Health Industry Development Institute (KHIDI); Ministry of Health & Welfare, Republic of Korea (grant number HC19C0215).
Kim et al. (16)	Korea(Samsung Medical Center and Asan Medical Center)	277 patients diagnosed with PTMC from 2007 to 2017, 234 patients were enrolled for AS;	Multicenter retrospective cohort study	The median follow-up duration was 51.0 (43.0–59.0) months	None reported
Lee et al. (17, 20) ^b^	Korea(Seoul National University Hospital, National Cancer Center, Seoul National University Bundang Hospital, and Borame Medical Center)	1182 patients with PTMC diagnosed by fine-needle aspiration or core needle biopsy, between 2016 and 2020 were screened, 755 patients were enrolled for AS;	Multicenter prospective cohort study	Mean duration of follow-up was 41.4 ± 16.0 months	Seoul National University Hospital (Research Grant 25-2016-0010); National Cancer Center (Research Grant 1810151–3 and 2210521-1).
Liu et al. (19)	China (Peking Union Medical College Hospital)	336 patients diagnosed with highly suspicious thyroid nodules by ultrasound and followed up by AS without immediate surgery from 2018 to 2021 were screened, 336 were enrolled for AS;	Prospective cohort study	The median follow-up duration was 28.5 (4.3–138) months	Non-profit Central Research Institute Fund of Chinese Academy of Medical Sciences (grant numbers: 2019XK320011).
Nagaoka et al. (15)	Japan (Cancer Institute Hospital and Nippon Medical School)	571 patients with PTMC diagnosed by US-guided FNAC are evaluated between 1995 and 2019 for AS;	Retrospective analysis	Mean duration of follow-up was 7.6 ± 5.0 years	JSPS KAKENHI Grant Number 20K08995
Yamamoto et al. (18)	Japan (Kuma Hospital)	4,632 patients aged over 20 years were diagnosed with PTMC (T1aN0M0) between February 2005 and December 2019 were evaluated, 2896 were enrolled for AS;	Cohort study	Median duration is 6.67 (1.02–17.6) years	None reported

PTC, papillary thyroid carcinoma; PTMC, papillary thyroid microcarcinoma; AS, active surveillance.

^a^Reported by authors in the published reference;

^b^Both references form one cohort were included in the analysis.

### Clinical characteristics

**Table 2** presents the characteristics of the study participants. The mean or median age ranged from 41.5 to 53.1 years, with a predominant inclusion of female patients (76.39%–87.80%). All the studies included patients with papillary microcarcinomas (<1.0 cm). Most studies included multifocal PTC, though three studies did not report this feature. Levothyroxine use varied significantly among studies. Excluding two studies that did not report this information, reports of TSH-suppressive therapy ranged from 0% to 46.3%. Most studies defined tumor progression as a diameter increase ≥3 mm or lymph node metastasis, with one study including a volume increase of ≥50% (16).

**Table 2:** Characteristics of study participants.

Source	Age at diagnosis	Female/Total (%)	Excluded larger PTCs^a^	Primary tumor size (mm)	Inclusion of multifocal PTC	Prevalence of LT4 usage (%)^b^	Clinical outcomes of Patients Undergoing AS
Hu et al. (13)	43.0 ± 11.1	297/386 (76.94)	Yes	7.1 ± 1.8	Yes	3.89	An increase in nodule size of 3 mm or more compared with the size at initiation of observation.
Jin et al. (14)	50.6 (43.0–58.6)	250/326 (76.68)	Yes	5.6 (4.4–6.8)	Yes	12.27	An increase in the maximal diameter of the nodule of 3 mm or more, and/or the development of new cervical LNM on US.
Kim et al. (16)	51.0 (43.0–59.0)	183/234 (78.21)	Yes	5.6 (4.4–6.7)	Not reported	0	A volume increase ≥50% or size increase ≥3 mm or new clinical LNM.
Lee et al (17, 20), ^c^	50 ± 12	534/699 (76.39)	Yes	6.2 ± 1.6	Not reported	not reported	A size increase of 3 mm or more in at least one-dimension, suspected extrathyroidal tumor extension, pathologic diagnosis of LNM, or suspected distant metastasis
Liu et al. (19)	43.7 ± 11.7	264/336 (78.57)	Yes	5.8 ± 1.9	Yes	0.89	An increase of 3 mm or more and subsequent FNAB confirming malignancy, or novel LNM or distant metastasis, or invasion of recurrent laryngeal nerve, trachea or esophagus
Nagaoka et al. (15)	53.1 ± 12.7	495/571 (86.69)	Yes	Unifocal 8.1 ± 2.2 Multifocal 8.3 ± 2.0	Yes	not reported	Tumor size enlargement ≥3 mm or development of LNM
Yamamoto et al. (18)	No LT4 group 57.22 ± 13.40 LT4 group 53.25 ± 13.82	No LT4 group 1615/1,901 (84.96); LT4 group 252/287 (87.80)	Yes	No LT4 group 6.78 ± 1.79 LT4 group 7.00 ± 1.74	not reported	12.83	Tumor enlargement (≥3 mm) and/or the novel appearance of lymph node metastasis.

PTC, papillary thyroid carcinoma; LT4, levothyroxine; AS, active surveillance; LNM, lymph node metastasis; US, ultrasonography; FNAB, fine-needle aspiration biopsy.

^a^The maximal diameter of the PTC is larger than 10 mm;

^b^Reported by authors in the published reference;

^c^Both references form one cohort were included in the analysis.

### Results of the meta-analyses

**Figure 2:**
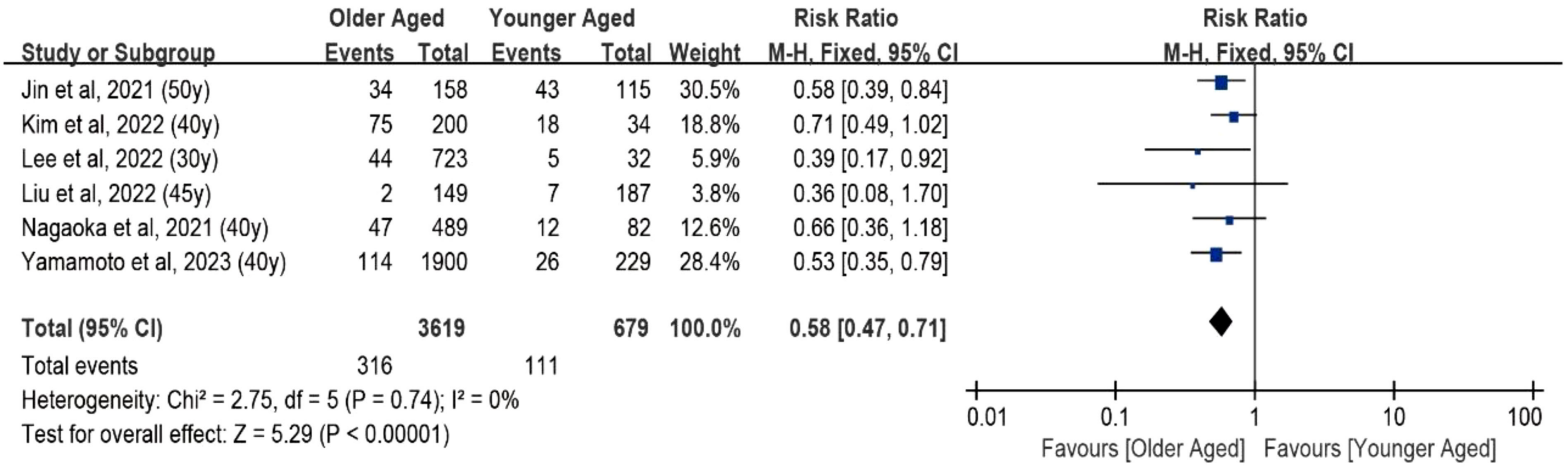
Forest plot of pooled risk ratios of tumor enlargement relative to age. Forest plot of the age-specific risk of a tumor progression (a size increase of 3 mm or more in at least one dimension, lymph node metastasis, or distant metastasis). Age was the cut point used in the references included in the analysis.

The fixed-effects meta-analysis examined the impact of aging on tumor progression under AS by incorporating data from six studies, totaling 4,725 patients. The pooled Risk Ratio (RR) for tumor growth of ≥3 mm in maximal diameter or lymph node metastasis in older adults (aged over 30–50 years) compared with younger individuals was 0.58 (95% CI, 0.47–0.71) (**Figure 2**). No statistically significant heterogeneity was observed in this study (I² = 0, df = 5, P = 0.74).

**Figure 3:**
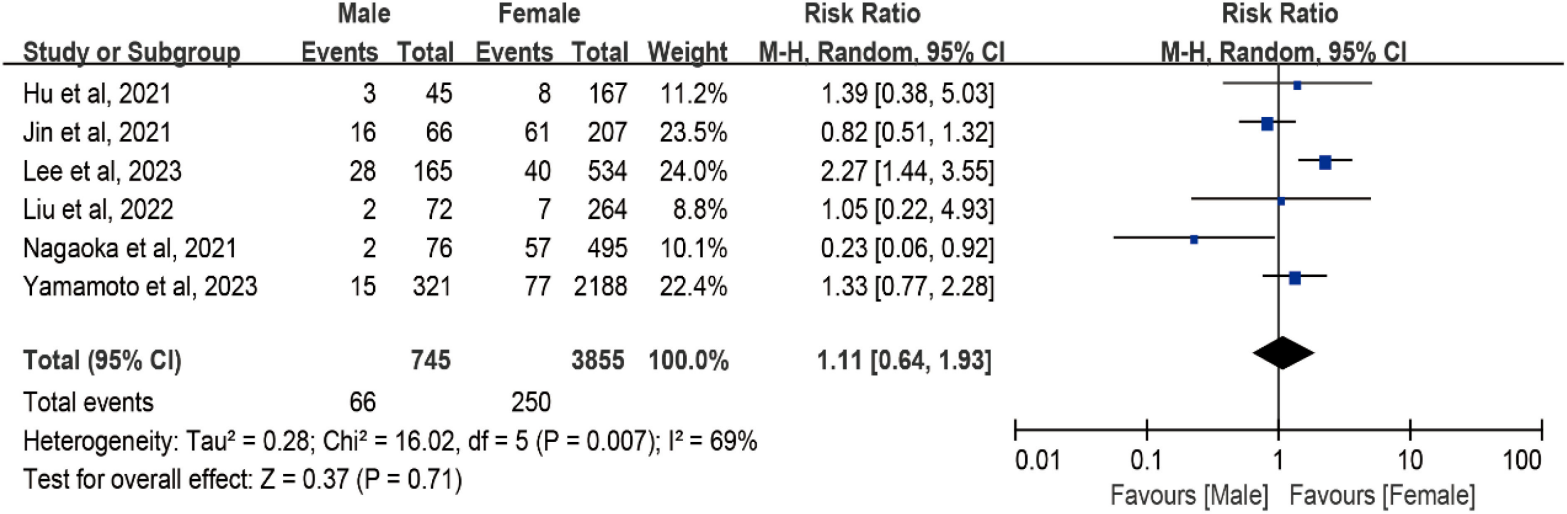
Forest plot of pooled risk ratios of tumor enlargement relative to sex. Forest plot of the gender-specific risk of a tumor progression (a size increase of 3 mm or more in at least one dimension, lymph node metastasis, or distant metastasis).

In the meta-analysis assessing the impact of sex on tumor progression in AS, data from six studies with a total of 4,916 patients were included. However, a relatively high level of heterogeneity was identified among the studies (I² = 69%; df = 5; P = 0.007). Consequently, a random effects meta-analysis was conducted. As illustrated in **Figure 3**, for male patients, the pooled risk ratio for tumor progression compared with female individuals was 1.11 (95% CI, 0.64–1.93; 4,916 patients, six studies).

Considering the limited number of included studies, further sensitivity analyses exploring subgroup effects were not conducted. Furthermore, due to the inclusion of fewer than 10 studies, an analysis of publication bias could not be meaningfully interpreted and was therefore not performed.

## Discussion

In the 1990s, advancements in detection techniques revealed that most low-risk PTMC exhibited latent, slow, or no progression (24–26). Kuma Hospital (Kobe, Japan) pioneered AS as an alternative approach for low-risk PTMC in 1993 (12), followed by the Cancer Institute Hospital (Tokyo, Japan), adopting a similar concept in 1995 (27). Over time, these two institutions have contributed significantly to our knowledge of this subject.

By 2010, after a decade of follow-up, the safety and benefits of AS had been substantiated. This pivotal evidence led to the Japanese Association of Endocrine Surgeons and the Japanese Society of Thyroid Surgeons publishing the first edition of the guidelines, endorsing AS as a viable management option for low-risk PTMC (28). Five years later, the American Thyroid Association (ATA) also recommended AS as a strategy for managing low-risk PTMC (29). However, it is essential to note that the prevalence of disease progression under AS varies significantly during follow-up across different cohorts, ranging from 6.5% to 39.7% (16, 17). The variation in the risk of tumor progression under AS prompted the exploration of influencing risk factors. The roles of aging and sex remain debatable.

Our study incorporated data from eight cohorts of PTC patients undergoing AS, revealing a reduced risk of tumor enlargement (defined as both a maximal diameter increase of ≥3 mm, a tumor volume increase of ≥50%, and/or the development of new lymph node metastasis from baseline) with advancing age. The older the low-risk PTC was diagnosed, the lower the risk of tumor progression. However, the risk of surgical complications of thyroidectomy significantly increases with age. Considering both the economic burden and impact on the quality of life of patients caused by surgery and postoperative complications, this dual consideration becomes a compelling factor for doctors when making decisions regarding the management of low-risk PTMC in elderly individuals.

Thyroid cancer (TC) incidence shows a strong sex difference, with most populations having an incidence that is about three times higher in female than in male; the same trend was also observed in PTC (30). I Interestingly, the mortality rate of TC shows less disparity between sexes (1). Some studies have identified male sex as a factor positively associated with poor outcomes in patients with surgically treated PTC (31), while others have different perspectives (32, 33). The results varied among the AS groups. In our study, although a higher prevalence was found in the female group, no difference in tumor progression under AS was observed between sexes. This provides solid evidence for decision-making regarding low-risk PTC in both males and females, indicating that sex should not be a significant factor. Neither male nor female patients exhibited a higher risk of tumor progression during AS.

### Strengths and limitations

The strengths of this study include a thorough electronic database search conducted by an experienced specialist, independent duplicate reviews for study selection, and meticulous appraisal of the data from the included studies.

However, limitations include the small number of available studies and patients, limited follow-up periods in some studies, challenges in evaluating publication bias or subgroup effects due to scarce data, constrained statistical power for detecting heterogeneity, and limited exploration of gray literature.

### Conclusion

In this systematic review and meta-analysis, we found that older adults (aged 30–50 years) may have a reduced risk of progression in papillary thyroid microcarcinoma (PTMC) under active surveillance (AS). No significant differences in progression risk were observed between sexes. This study focused on PTMC and provided robust evidence to support clinical decision-making in the management of low-risk PTMC. These findings underscore the importance of individualized treatment strategies to achieve optimal outcomes in each patient.
